# Multimorbidity Extent and Contributing Factors Among Hospitalized Chronic Kidney Disease Patients in Resource‐Limited Settings

**DOI:** 10.1155/ijne/7195511

**Published:** 2026-11-14

**Authors:** Tirsit Ketsela Zeleke, Anteneh Amdu Ferede, Fasil Bayafers Tamene, Liknaw Workie Limenh, Samuel Agegnew Wondm, Rahel Belete Abebe, Ousman Abubeker Abdela

**Affiliations:** ^1^ Department of Pharmacy College of Health Sciences Debre Markos University Debre Markos Ethiopia dmu.edu.et; ^2^ Department of Internal Medicine College of Medicine and Health Sciences Debre Markos University Debre Markos Ethiopia dmu.edu.et; ^3^ Department of Pharmaceutics School of Pharmacy College of Medicine and Health Sciences University of Gondar Gondar Ethiopia uog.edu.et; ^4^ Pharmacy Education and Clinical Services Directorate College of Health Sciences Debre Tabor University Debre Tabor Ethiopia dtu.edu.et; ^5^ Department of Clinical Pharmacy School of Pharmacy College of Medicine and Health Sciences University of Gondar Gondar Ethiopia uog.edu.et

**Keywords:** associated factors, chronic kidney disease, Ethiopia, hospitalized patient, multimorbidity

## Abstract

**Background:**

Chronic kidney disease (CKD) is frequently accompanied by multimorbidity, resulting in increased hospitalization, healthcare expenditure, and medication burden. Despite this, evidence on the extent and contributing factors of multimorbidity among CKD patients in Ethiopia is scarce. This study assessed the prevalence of multimorbidity and its associated factors among hospitalized CKD patients.

**Methods:**

A hospital‐based cross‐sectional study was conducted among hospitalized CKD patients in three comprehensive specialized hospitals. Participants were selected using simple random sampling. Descriptive statistics were used to summarize the data, and comparisons were made using the chi‐square test and the *t*‐test. Factors associated with multimorbidity were identified using binary logistic regression. Statistical significance was defined as a *p* value of less than 0.05 with a 95% confidence interval.

**Results:**

The prevalence of multimorbidity was 85.7%, with participants having an average of two comorbid conditions. Hypertension was the most common comorbid condition. Multimorbidity was significantly associated with aging (AOR = 1.67 (95% CI 1.21–2.31), higher body mass index (AOR = 1.70 (95% CI 1.23–2.35), and lower creatinine clearance (AOR = 0.95 (95% CI 0.92–0.99)).

**Conclusion:**

Multimorbidity is highly prevalent among hospitalized CKD patients in Ethiopia. Aging, increased body mass index, and reduced renal function were significant contributing factors. Clinical management strategies should prioritize early detection and tailored care for elderly and overweight patients and those with impaired kidney function.

## 1. Introduction

Chronic kidney disease (CKD) is a progressive decline in kidney function, regardless of the underlying cause, defined by a creatinine clearance (estimated glomerular filtration rate) of less than 60 mL/min/1.73 m^2^ or the presence of kidney damage for at least 3 months [1]. CKD is a major global public health concern, affecting more than 800 million people worldwide [2] and contributing substantially to healthcare costs, morbidity, and mortality [3]. Patients with CKD frequently experience a wide range of nutritional and metabolic disturbances that predispose them to multiple chronic conditions [4]. Common comorbidities include anemia, hypertension, cardiovascular disease, bone and mineral disorders, and diabetes mellitus [5, 6]. The coexistence of two or more chronic diseases in an individual is referred to as multimorbidity [7], which is strongly associated with poorer clinical outcomes, increased hospitalizations, reduced quality of life, and higher healthcare expenditures [8, 9].

Multimorbidity among CKD patients extend beyond physical comorbidities. Mental health disorders such as anxiety and depression are increasingly recognized in this population [10, 11]. These conditions negatively influence self‐care, medication adherence, and disease progression, thereby increasing likelihood of additional comorbidities [12, 13]. The CKD management is further complicated by frequent medication use to treat both renal impairment and coexisting chronic illnesses [14, 15]. Consequently, patients commonly experience multiple drug use [16–18], which increases the risk of medication errors, adverse drug effects, and drug‐drug interactions, making pharmacological management particularly challenging since many of these drugs require renal dose adjustment [19]. Therefore, it is beneficial to know which comorbidities are typical among CKD patients to take appropriate management and prevention measures. Additionally, such studies may be used to determine the best treatment for each individual, customize therapies, lower the utilization of healthcare services, and efficiently allocate expenditures [20, 21]. Minimizing therapeutic load, such as numerous visits or polypharmacy, is also crucial to enhancing healthcare in CKD patients, where multimorbidity frequency is high [6, 22, 23].

Although multimorbidity among CKD patients has been widely studied in high‐income countries, evidence from resource‐limited settings, including sub‐Saharan Africa, remains scarce [24]. Multimorbidity patterns and contributing factors may differ in low‐resource environments due to delayed healthcare‐seeking behavior, limited diagnostic capacity, late presentation to health facilities, and different disease epidemiology [25, 26]. Few studies from regions such as India and Brazil have examined multimorbidity in CKD patients [27, 28]; however, no multicenter study has investigated the extent and contributing factors of multimorbidity among hospitalized CKD patients in Ethiopia. Generating such context‐specific evidence is critical to inform clinical management and resource allocation in similar low‐resource settings.

Despite the well‐recognized clinical and economic burden associated with multimorbidity in CKD, little is known about its prevalence, pattern, and contributing factors in Ethiopia and across similar low‐resource settings. Existing evidence on CKD multimorbidity is predominantly derived from high‐income countries, where early disease detection, specialized nephrology care, and well‐established diagnostic systems facilitate comprehensive identification of comorbid conditions. In contrast, healthcare systems in Ethiopia face challenges such as delayed healthcare‐seeking behavior, limited availability of diagnostic tests, and fragmented documentation of chronic conditions, which may lead to under‐recognition or delayed diagnosis of comorbidities. To date, no multicenter study has investigated multimorbidity among hospitalized CKD patients in Ethiopia, a subgroup likely to represent the most clinically complex patients and for whom multimorbidity presents critical implications for treatment decisions, resource use, and clinical outcomes. Evidence from such settings is essential to understand whether the drivers and patterns of multimorbidity differ from those reported in high‐income contexts and to inform context‐appropriate clinical management strategies.

This study represents the first multicenter investigation to comprehensively assess the extent and contributing factors of multimorbidity among hospitalized patients with CKD in Ethiopia. It provides novel, context‐specific evidence from a resource‐limited setting where such data remain scarce, particularly as existing multimorbidity research is predominantly derived from high‐income countries with more advanced healthcare systems and diagnostic capacity [24]. By addressing this gap, the study will contribute to a better understanding of multimorbidity in underrepresented populations and supports the development of context‐appropriate clinical and public health strategies. Therefore, this study aimed to assess the extent of multimorbidity and identify its contributing factors among hospitalized CKD patients in Ethiopia, providing novel and context‐specific evidence to guide clinical practice, resource planning, and future research in low‐resource healthcare environments.

## 2. Methods

### 2.1. Study Setting and Design

A hospital‐based cross‐sectional study was conducted from August 10, 2022, to July 30, 2023, in Northwest Ethiopia. In this region, there are six comprehensive, publicly specialized hospitals that provide tertiary‐level care for patients with CKD. From these eligible facilities, three hospitals were selected using a simple random sampling method to ensure representativeness and minimize selection bias. The selected hospitals were the University of Gondar Comprehensive Specialized Hospital, Felege Hiwot Comprehensive Specialized Hospital, and Debre Markos Comprehensive Specialized Hospital.

All three hospitals function as public referral centres serving large catchment populations and provide inpatient care for patients with CKD. Although the hospitals differ in operational characteristics such as catchment population size, bed capacity, and patient volume, with the University of Gondar and Felege Hiwot serving as larger tertiary referral centres and Debre Markos serving a comparatively smaller population, they share similar healthcare system constraints. These include limited availability of advanced diagnostic technologies, reliance on routine laboratory investigations, delayed patient presentation, and restricted access to specialized nephrology services and multidisciplinary care teams.

### 2.2. Source and Study Population

The source population consisted of all adult patients with CKD admitted to the three hospitals during the study period. The study population included hospitalized CKD patients who were available during the data collection period.

### 2.3. Eligibility Criteria

Patients aged 18 years and older were enrolled in the study. Individuals with incomplete clinical data or those unable to communicate were excluded from the study.

### 2.4. Sample Size Determination

Single population proportion formula was used to estimate the sample, where the following assumptions were considered: 50% prevalence, 95% confidence interval, and 5% margin of error.

(1) n=Za/22pqd2,

where *Z* statistics (at 95% CI = 1.96), *p* (population proportion), and *d* (margin of error).

(2) n=1.962x0.50.5x0.052=384.

However, according to the previous hospital record of CKD cases, the total number of patients admitted annually was 571, which was fewer than 10,000. Given the relatively low prevalence of CKD at the study sites, we applied a population correction formula to refine the final sample size. *e*. As a result, we calculated our final sample size using population correction.

Population correction formula

(3) nf=n1+n/N,

where *N* = total number of patients who could be presented. *n* = sample size before population correction. *n* *f* = final sample size.

(4) nf=3841+384571/=230.

Therefore, our final sample size was **230**.

### 2.5. Sampling Technique

Patients from each hospital were enrolled using proportional allocation. The three hospitals were selected using a simple random sampling method, and participants were selected at random from each institution using a simple random selection process.

### 2.6. Study Variables

Multimorbidity was the dependent variable. While sociodemographic parameters (sex, age, residence, educational level, occupation, monthly income, and smoking history) and medical‐related parameters (body mass index [BMI], creatinine clearance value, serum creatinine value, type of comorbid disease, number of comorbidities, and presence of dialysis) were included as independent variables.

### 2.7. Operational Definition

Multimorbidity was defined as the presence of two or more chronic diseases in the same individual [7]. It is the most applied standard in multimorbidity research and enables comparability across international studies. The threshold of at least two chronic conditions is widely accepted in epidemiological research, including studies published in high‐impact journals and in frameworks proposed by the World Health Organization. Although alternative definitions exist, such as requiring three or more chronic conditions or using weighted comorbidity indices, these approaches may underestimate multimorbidity in populations with a high burden of chronic illness, such as patients with CKD.

In this study, chronic diseases contributing to multimorbidity were identified through patient self‐report and subsequently verified using physician diagnoses, medical records, laboratory findings, and medication profiles. Comorbid conditions considered included, but were not limited to, hypertension, diabetes mellitus, dyslipidemia, ischemic heart disease, heart failure, cardiac arrhythmias, peripheral vascular disease, stroke, anxiety or depression, musculoskeletal disorders such as osteoarthritis or rheumatoid arthritis, endocrine and metabolic disorders including thyroid dysfunction and obesity, chronic hepatic disease, and malignancy. These conditions were included based on their clinical relevance, common occurrence in CKD patients, and the feasibility of objective verification within routine hospital records.

### 2.8. Data Collection Tools and Procedure

A structured questionnaire was developed after reviewing a variety of literature sources. Four trained nurses collected the data under supervision of two clinical supervisors. The data collection instrument had two sections: Sociodemographic characteristics were obtained through interviews, and clinical/medical findings were retrieved from medical records. Some parameters, such as weight and height, were not usually recorded; in this case, the findings were obtained through measurement, and BMI was calculated using the formula, BMI = weight in kg/height in meters^2^ [29]. Other laboratory tests, including serum creatinine, were taken from the medical charts of the patients, and the estimated creatinine clearance was calculated using the Cockcroft–Gault formula [30]. Moreover, the stage of the disease was classified based on estimated creatinine clearance. Although other creatinine clearance estimation equations such as MDRD and CKD‐EPI are recommended in current clinical guidelines, the Cockcroft–Gault equation remains the routine method used in the participating hospitals. This is because it aligns with existing clinical practice, and the required variables (age, serum creatinine, and weight) are consistently documented in patients’ medical records. We acknowledge that Cockcroft–Gault may have limitations, including reduced accuracy in older adults or in individuals with very high or very low body weight; however, it was the most feasible formula within the operational constraints of the study settings. Regarding the assessment of the presence of comorbid conditions, multimorbidity was determined by first asking patients to self‐report their known chronic conditions. To ensure accuracy, each reported condition was verified using physician diagnoses, medical charts, laboratory results, and medication profiles. For instance, a prescription of antihypertensive drugs or reviews of increased blood pressure from medical records were used to confirm the presence of hypertension. A list of comorbidities was selected based on their wide range of availability in individuals with CKD and their straightforwardness of detection from medical data and patient self‐report. The comorbid conditions chosen consisted of cerebrovascular illness (stroke), hypertension, anxiety, depression, or both (or other mental health issues), dyslipidemia, heart disease (heart attack, cardiac arrhythmias, ischemic heart disease, and atrial fibrillation), diabetes, peripheral vessel diseases, muscular and skeletal diseases, malignancy, hepatic disease, and others. Finally, an assessment of multimorbidity has been done based on previous literature [7].

### 2.9. Data Processing and Analysis

After accuracy and uniformity checks, the data were loaded into Epi‐data 4.6 and exported to SPSS 26 for additional analysis. For variables with categories, computations of frequency and percentage were performed, while the continuous variables were analyzed using the mean and standard deviation. The proportions and means were compared using the chi‐square test and the *t*‐test, respectively. An analysis of the potential factors of multimorbidity was conducted with a binary logistic regression model. The Hosmer and Lemeshow goodness‐of‐fit test was used to assess the model’s fitness, and in order to identify and eliminate redundant variables that can affect our estimate, the multicollinearity of the independent variables was analyzed using the variance inflation factor, which was within an acceptable level of one to five. In the bivariable analysis, variables with *p* values less than 0.25 were included in the multiple binary logistic regression model. In addition to *p* value screening, variables were also selected based on clinical relevance and supporting evidence from previous literature to ensure that important predictors were not excluded solely on statistical grounds. The adjusted odds ratio was used to report the statistical association. A significant association with the outcome variable, a *p* value of less than 0.05, was used. While *p* value‐based selection remains a widely used approach in epidemiological research, we recognize its drawbacks, particularly the risk of excluding clinically important variables that may not meet the statistical significance threshold. To address this concern, we selected variables not only based on statistical significance but also on their clinical relevance and prior evidence from the literature. Additionally, we assessed multicollinearity to ensure the robustness of our model.

Finally, texts, tables, and figures were utilized to show the study’s findings.

### 2.10. Data Quality Assurance

To ensure consistency, the investigator developed the questionnaire in English. Amharic and English‐speaking staff then translated it both forward and reverse. The translation indicated that the instrument needed to be adjusted. The lead investigator of the study trained supervisors and data collectors on the objectives, methods, and ethical aspects of the study. At Tibebe Ghion Specialized Hospital, a pretest was performed to assess the cultural acceptability and clarity of the questionnaire. Moreover, the supervisors offered on‐site supervision throughout the duration of the data collection process, and the investigator assessed and confirmed the data for consistency and completeness.

## 3. Results

### 3.1. Sociodemographic Parameters of the Study Participants

A total of 230 patients were included in this study. The mean age of the participants was 48.84 years old, with a standard deviation of ±14.90 years. More than half of the participants (55.2%) were male, and 51.7% of the respondents resided in urban areas. Regarding educational status, 29.6% of participants had attained a college or higher education level. In terms of occupation, 30.9% were government employees. The average monthly income of participants was 2640.86 Ethiopian birr (ETB) (Table 1).

**TABLE 1:** Sociodemographic characteristics of hospitalized CKD patients at specialized comprehensive hospitals, Amhara, Ethiopia.

**Variables**	**Frequency (*n*)**	**Percent (%)**

Sex		
Male	127	55.2
Female	103	44.8
Residence		
Rural	111	48.3
Urban	119	51.7
Education status		
No formal education	61	26.5
Primary education	42	18.3
Secondary education	59	25.7
College and above	68	29.6
Occupation		
Farmer	58	25.2
Housewife	34	14.8
Government employee	71	30.9
Private worker	56	24.3
Other^∗^	11	4.8

	**Mean**	**Standard deviation**

Age	48.84	±14.90
Monthly income in ETB	2640.86	±1794.33

^∗^Pensioner, daily laborer, student. ETB: Ethiopian birr.

### 3.2. Medical Related Parameters of the Study Participants

The average BMI of the participants was 25.10 kg/m^2^ with a standard deviation of ±3.43. Regarding CKD staging, 23.0% of the patients were classified as having stage five CKD. Laboratory recordings showed that the mean level of serum creatinine level was 3.35 mg/dL, while the mean creatinine clearance was 42.98 mL/min. Moreover, 23.0% of the patients were receiving dialysis treatment (Table 2).

**TABLE 2:** Medical related parameters among hospitalized CKD patients at specialized comprehensive hospitals, Amhara, Ethiopia.

**Variable**	**Frequency (*n*)**	**Percent (%)**

Smoking history		
Currently smoker	20	8.7
Previous smoker	26	11.3
Never smoke	184	80.0
Stage of the disease		
Stage one	49	21.3
Stage two	43	18.7
Stage three	38	16.5
Stage four	47	20.4
Stage five	53	23.0
Dialysis therapy		
Absent	177	77.0
Present	53	23.0

	**Mean**	**Standard deviation**

BMI	25.10 kg/m^2^	±3.43
Serum creatinine level	3.35 mg/dL	±2.85
Estimated creatinine clearance	42.98 mL/min	±30.25

Abbreviation: BMI, body mass index.

### 3.3. Extent of Multimorbidity

The prevalence of multimorbidity among the study participants was 85.7%. The prevalence of multimorbidity was consistently high across all three hospitals, with no statistically significant differences observed between facilities. Regarding the pattern of comorbid conditions, a total of 557 multimorbidity cases were recorded, with an average of two comorbid conditions per patient. Hypertension was the most reported comorbid condition, accounting for 22.4% of cases, followed by diabetes mellitus (16.5%) and dyslipidemia (14.2%) (Table 3). Additionally, more than half of the patients (55.8%) had a minimum of two comorbid conditions (Figure 1).

**TABLE 3:** Extent of multimorbidity among hospitalized CKD patients at specialized comprehensive hospitals, Amhara, Ethiopia.

Variable		Frequency (*N*)	Percent (%)
Multimorbidity	Absent	33	14.3
	Present	197	85.7

Type of the comorbid condition	Hypertension	125	22.4
	Diabetics Mellitus	92	16.5
	Congested Heart Failure	43	7.8
	Dyslipidemia	79	14.2
	Acute Coronary Syndrome	37	6.6
	Stroke	39	7.0
	Asthma	19	3.4
	Atrial Fibrillation	33	6.0
	Osteoporosis	9	1.6
	Peptic Ulcer Disease	51	9.2
	Tuberculosis	11	2.0
	Cirrhosis	7	1.2
	RVI	4	0.7
	Others^∗^	8	1.4
	Total	557	100

		**Mean**	**Standard deviation**

Average number Of comorbidities		2.42	±1.04

^∗^Arthritis, epilepsy, depression, chronic obstructive pulmonary disease.

**FIGURE 1:**
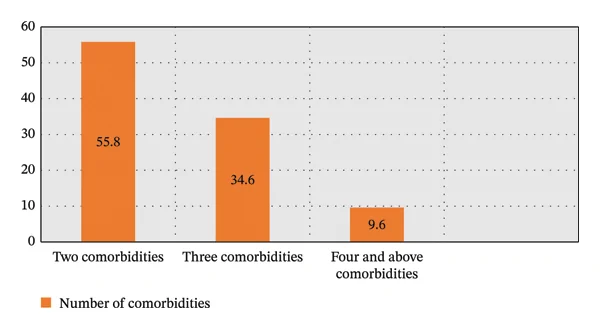
Distribution of multimorbidity among hospitalized CKD patients in specialized comprehensive hospitals, Amhara, Ethiopia.

## 4. Contributing Factors of Multimorbidity

The results of the binary logistic regression analysis demonstrated that aging, increased BMI, and a reduced creatinine clearance were significantly associated with multimorbidity. However, medical‐related factors such as CKD stage, smoking history, and dialysis therapy did not show a significant association. Similarly, sociodemographic factors including sex, residence, education, occupation, and monthly income were not significantly associated with multimorbidity. As the participants age older, the odds of multimorbidity increase by 1.67 times (AOR = 1.67 [95% CI 1.21–2.31]). The risk of multimorbidity increased by 1.70 when patients’ BMI increased (AOR = 1.70 [95% CI 1.23–2.35]), and as the creatinine clearance value increased, the vulnerability to multimorbidity decreased by 0.95 times (AOR = 0.95 [95% CI 0.92–0.99]). Moreover, a sensitivity analysis excluding patients on maintenance hemodialysis showed that the direction and magnitude of associations between multimorbidity and key predictors remained consistent (Table 4).

**TABLE 4:** Contributing factors of multimorbidity vulnerability among hospitalized CKD patients at specialized comprehensive hospitals, Amhara, Ethiopia.

Variables	Multimorbidity		Bivariable analysis			Multivariable analysis		
	Yes	No	*p* value	COR	CI	*p* value	AOR	CI
Age	52.39 ± 13.04	27.70 ± 3.10	< 0.00	1.43	1.24–1.66	< 0.00**^∗^**	1.67	1.21–2.31
Sex								
Male	113	14	0.11	1.82	0.86–3.84	0.81	1.30	0.14–11.48
Female	84	19		1			1	
Residence								
Rural	91	20	0.12	0.55	0.26–1.18	0.80	1.29	0.16–10.38
Urban	106	13		1			1	
Education status								
No formal education	53	8	0.98	1.01	0.36–2.80			
Primary level	35	7	0.62	0.76	0.26–2.22			
Secondary level	50	9	0.74	0.84	0.31–2.29			
College and higher level	59	9		1				
Occupation								
Farmer	47	11	0.95	0.94	0.17–5.02			
Housewife	31	3	0.40	2.29	0.33–15.93			
Government employee	61	10	0.72	1.35	0.25–7.21			
Private worker	49	7	0.61	1.55	0.27–8.72			
Other	9	2		1				
Smoking history								
Currently smoker	17	3	0.80	0.85	0.23–3.12	0.33	8.60	0.10–68.55
Previously smoker	20	6	0.17	0.50	0.18–1.37	0.17	0.08	< 0.00–2.90
Never smoke	160	24		1			1	
Stage of the disease								
Stage one	42	7		1				
Stage two	37	6	0.96	1.02	0.31–3.33			
Stage three	31	7	0.60	0.73	0.23–2.32			
Stage four	40	7	0.93	0.95	0.30–2.95			
Stage five	47	6	0.65	1.30	0.40–4.19			
Dialysis therapy								
Absent	151	26		1				
Present	46	7	0.78	0.88	0.36–2.16			
BMI	25.88 ± 2.60	20.42 ± 4.10	< 0.00	1.68	1.44–1.96	< 0.00**^∗^**	1.70	1.23–2.35
Creatinine clearance	35.96 ± 24.59	71.57 ± 20.12	< 0.00	0.94	0.93–0.96	0.02**^∗^**	0.95	0.92–0.99

*Note:* Bold texts indicate variables that are significantly associated with the outcome.

^∗^Statistically significant. AOR: adjusted odds ratio, BMI: body mass index, COR: crud odds ratio, CI: confidence interval.

## 5. Discussion

To the best of our knowledge, this is the first study in Ethiopia to assess the extent of multimorbidity and associated factors among hospitalized patients with CKD. Unlike the extensive body of multimorbidity literature from high‐income countries, evidence from low‐resource healthcare systems remains limited. In such settings, diagnostic constraints, delayed health‐seeking behavior, late CKD presentation, and limited access to specialist care may influence the detection and documentation of comorbid conditions. By using data from three hospitals, this study provides context‐specific evidence from a resource‐constrained health system and contributes to closing an important knowledge gap.

More than half of participants in this study were male, consistent with findings from tertiary hospitals in Eastern India [27]. The higher proportion of male CKD patients may be attributed to lifestyle‐related factors such as increased alcohol intake, smoking, unhealthy dietary habits, inadequate physical activity, and reduced engagement in preventive healthcare, which have been reported as contributing to chronic illnesses. In terms of CKD staging, a majority of patients had stage 5 CKD, similar to findings from India [31]. Conversely, another study reported a predominance of Stage 3 CKD [32]. The discrepancy in the distribution of CKD stages may be due to variations in diagnostic practices, delayed detection, or differences in serum creatinine‐based estimation formulas used. Nearly all stage 5 CKD patients in our study were undergoing dialysis treatment, whereas conservative management was more common in another setting [32], suggesting that differences in resource availability and treatment protocols may influence care pathways.

A key finding of this study is the high prevalence of multimorbidity (≥ 2 chronic conditions) among hospitalized CKD patients. In our study, we used the widely accepted definition of multimorbidity as the presence of at least two chronic conditions in an individual, which aligns with standard epidemiological and clinical research practices. This approach allows for comparability with previous studies and facilitates the identification of key patterns in multimorbidity among hospitalized CKD patients. This aligns with studies reporting similarly high multimorbidity rates in CKD patients across different regions [6, 27, 33, 34]. Slight variations in prevalence between studies may reflect differences in study design, CKD stages included, and the number and types of comorbidities assessed.

The kidneys play a central role in maintaining electrolyte balance, removing metabolic waste, and supporting endocrine function. As CKD progresses, accumulation of toxins, electrolyte disturbances, and hormonal dysregulation occur, triggering systemic metabolic changes. These pathophysiological processes contribute to the development of multiple comorbidities, particularly hypertension, diabetes mellitus, and lipid disorders [22]. These diseases are both risk factors for CKD development and consequences of renal impairment, creating a bidirectional relationship that drives multimorbidity.

Regarding the pattern of multimorbidity, hypertension was the most frequently identified comorbidity, consistent with previous studies [35–37]. The coexistence of hypertension and CKD is well established. Reduced sodium excretion, activation of the renin–angiotensin–aldosterone system, and reduced creatinine clearance contribute to blood pressure elevation [38]. CKD is also closely linked with cardiovascular diseases and diabetes [33, 39], and the presence of multiple chronic diseases in CKD patients increases mortality risk and worsens quality of life [6].

The present study also explored the potential factors contributing to multimorbidity, which consisted of aging, increased BMI, and a decreased creatinine clearance. Identifying these contributing factors is crucial for implementing preventive strategies. Aging was found to be significantly associated with higher likelihood of multimorbidity, which is consistent with global evidence. Adults of all ages can be multimorbid; however, as people age, their comorbid diseases tend to become more frequent and complicated [40]. A systematic review reported that the rate of multimorbidity increases as people age [41]. Similarly, higher BMI was positively associated with multimorbidity, consistent with findings from Brazil [42]. A rise in BMI is linked to the development of obesity and overweight, which play a role in the development of comorbid conditions, resulting in a higher mortality rate as well as a number of medical problems, including stroke and cardiovascular disease [43, 44]. Another factor contributing to multimorbidity in CKD patients was a decline in kidney function. Interestingly, decreased creatinine clearance was associated with lower odds of multimorbidity, a finding similar to a cohort study conducted in Japan [45]. Although this observation may initially seem counterintuitive, survival bias provides a plausible explanation. Patients with advanced CKD and severely reduced creatinine clearance have a substantially increased risk of mortality and, therefore, may not survive long enough to accumulate multiple chronic conditions. Additionally, clinical care in advanced CKD often prioritizes life‐preserving renal management (e.g., dialysis initiation and management of electrolyte imbalances). As a result, routine screening or investigation for other chronic diseases may receive lower priority, which can limit the diagnosis or documentation of comorbid conditions. Consequently, patients with very low creatinine clearance may appear to have less comorbidity not because they are healthier, but because they have a shorter survival window or their care is focused primarily on renal complications. Several factors may explain this apparent protective association. First, patients with more severe CKD, characterized by markedly reduced creatinine clearance, may have a higher mortality rate and, therefore, may not survive long enough to develop multiple comorbidities. Second, multimorbidity is often associated with increased healthcare engagement, leading to earlier detection and management of CKD in patients with multiple comorbid conditions. In contrast, patients with advanced CKD may have their care primarily focused on renal management, with less emphasis on diagnosing and documenting additional comorbidities. Third, the complex interplay between CKD progression and multimorbidity may vary among patient subgroups. For instance, while conditions such as diabetes and hypertension are strong risk factors for CKD development, once CKD reaches an advanced stage, renal complications may become the primary concern, potentially overshadowing the presence or clinical recognition of other comorbidities. A sensitivity analysis excluding patients on maintenance hemodialysis was conducted to assess whether the inclusion of these patients influenced the study findings. The analysis demonstrated that the direction, magnitude, and statistical significance of the associations between multimorbidity and key predictors—age, BMI, and creatinine clearance—remained largely unchanged. This indicates that the observed relationships were not driven by patients undergoing dialysis and that the inclusion of these patients did not materially alter the results. These findings provide additional confidence in the robustness and generalizability of our results across both predialytic and dialytic CKD populations.

In the present study, there was no significant association between cigarette smoking and multimorbidity. This may be due to the relatively low number of participants with a history of smoking, suggesting that further studies with larger sample sizes are needed to explore this relationship. Finally, some associations in our multivariable model exhibited wide confidence intervals, which may indicate limited statistical power for certain predictors. This likely reflects the heterogeneity of the study population and reinforces the need for larger studies capable of detecting subtle but clinically meaningful associations.

### 5.1. Limitations

This study’s limitation is that it can be challenging to prove a cause‐and‐effect relationship because data is gathered for a cross‐sectional study design, one event at a time. Moreover, there is a potential for survival bias, as our study included only hospitalized CKD patients, which may have led to the underrepresentation of patients with more severe conditions who did not survive hospitalization or those with milder conditions who were not admitted. Future studies employing prospective designs with a larger and more diverse population are recommended to address these constraints.

### 5.2. Strengths

This is a multicenter study including data from three different settings, which enhances the generalizability of the findings. Additionally, patients with high BMI were provided with counseling on lifestyle modifications, such as regular exercise and appropriate dietary recommendations, contributing to the studies practical on patient care.

## 6. Conclusion

Hospitalized CKD patients exhibited a high prevalence of multimorbidity. Aging, increased BMI, and reduced creatinine clearance were identified as significant contributing factors. Older patients, those with impaired renal function, or those with high BMI should receive targeted attention from healthcare professionals to improve clinical outcomes. Furthermore, the findings of this study provide crucial insights for enhancing the prevention, early screening, and management of multimorbidity in CKD patients.

**Nomenclature**
BMI: Body mass index
CKD: Chronic kidney disease

## Author Contributions

Conceptualization: Tirsit Ketsela Zeleke, Anteneh Amdu Ferede, and Ousman Abubeker Abdela. Data curation: Tirsit Ketsela Zeleke. Formal analysis: Tirsit Ketsela Zeleke, Rahel Belete Abebe, Samuel Agegnew Wondm, Fasil Bayafers Tamene, and Ousman Abubeker Abdela. Investigation: Tirsit Ketsela Zeleke, Anteneh Amdu Ferede, Liknaw Workie Limenh, and Ousman Abubeker Abdela. Methodology: Tirsit Ketsela Zeleke, Rahel Belete Abebe, Samuel Agegnew Wondm, Liknaw Workie Limenh, and Ousman Abubeker Abdela. Project administration: Tirsit Ketsela Zeleke, Rahel Belete Abebe, Anteneh Amdu Ferede, Fasil Bayafers Tamene, and Ousman Abubeker Abdela. Resources: Tirsit Ketsela Zeleke, Anteneh Amdu Ferede, Samuel Agegnew Wondm, and Liknaw Workie Limenh. Software: Tirsit Ketsela Zeleke, Rahel Belete Abebe, and Fasil Bayafers Tamene. Supervision: Tirsit Ketsela Zeleke, Anteneh Amdu Ferede, Samuel Agegnew Wondm, and Fasil Bayafers Tamene. Validation: Tirsit Ketsela Zeleke, Rahel Belete Abebe, Samuel Agegnew Wondm, Liknaw Workie Limenh, Fasil Bayafers Tamene, Anteneh Amdu Ferede, and Ousman Abubeker Abdela. Visualization: Tirsit Ketsela Zeleke, Fasil Bayafers Tamene, Rahel Belete Abebe, Liknaw Workie Limenh, and Ousman Abubeker Abdela. Writing–original draft: Tirsit Ketsela Zeleke, Anteneh Amdu Ferede, Fasil Bayafers Tamene, and Ousman Abubeker Abdela. Writing–review and editing: Tirsit Ketsela Zeleke, Rahel Belete Abebe, Anteneh Amdu Ferede, Samuel Agegnew Wondm, Liknaw Workie Limenh, and Ousman Abubeker Abdela.

## Funding

There is nothing to reveal about the funding.

## Ethics Statement

The study adhered to the Declaration of Helsinki, and each participant provided informed consent after being fully told about the research. Written informed consent was obtained from all study participants. Ethical clearance was obtained from the University of Gondar College of Medicine and Health Sciences’ ethical review committee with a reference number 5095/066. Additionally, to protect the confidentiality of the participants, no identifying information was included in the questionnaire.

## Conflicts of Interest

The authors declare no conflicts of interest.

## Data Availability

The manuscript contains all the required information. The corresponding author is able to provide any other information upon request.
